# Investigation of peripheral inflammatory biomarkers in association with violence in schizophrenia

**DOI:** 10.1186/s12888-024-05966-y

**Published:** 2024-07-31

**Authors:** Tao Yu, Wenzhi Pei, Chunyuan Xu, Xulai Zhang, Chenchen Deng

**Affiliations:** 1 Affiliated Psychological Hospital of Anhui Medical University; Anhui Mental Health Center; Hefei Fourth People’s Hospital; Anhui Clinical Research Center for mental disorders, Hefei, Anhui 230022 China; 2 Hefei Maternity & Child Health Hospital, Hefei, Anhui 230022 China

**Keywords:** Schizophrenia, Violence, Inflammation

## Abstract

**Background:**

Violent behavior carried out by patients with schizophrenia (SCZ) is a public health issue of increasing importance that may involve inflammation. Peripheral inflammatory biomarkers, such as the systemic immune inflammation index (SII), the neutrophil lymphocyte ratio (NLR), the platelet-lymphocyte ratio (PLR) and the monocyte lymphocyte ratio (MLR) are objective, easily accessible and cost-effective measures of inflammation. However, there are sparse studies investigating the role of peripheral inflammatory biomarkers in violence of patients with SCZ.

**Methods:**

160 inpatients diagnosed with SCZ between January and December 2022 were recruited into this study. Violent behavior and positive symptoms of all participants were evaluated using Modified Overt Aggression Scale (MOAS) and Positive and Negative Syndrome Scale (PANSS), respectively. The partial correlation analysis was performed to examine the relationship of inflammatory indices and positive symptoms. Based on machine learning (ML) algorithms, these different inflammatory indices between groups were used to develop predictive models for violence in SCZ patients.

**Results:**

After controlling for age, SII, NLR, MLR and PANSS positive scores were found to be increased in SCZ patients with violence, compared to patients without violence. SII, NLR and MLR were positively related to positive symptoms in all participants. Positive symptoms partially mediated the effects of peripheral inflammatory indices on violent behavior in SCZ. Among seven ML algorithms, penalized discriminant analysis (pda) had the best performance, with its an area under the receiver operator characteristic curve (AUC) being 0.7082. Subsequently, with the use of pda, we developed predictive models using four inflammatory indices, respectively. SII had the best performance and its AUC was 0.6613.

**Conclusions:**

These findings suggest that inflammation is involved in violent behavior of SCZ patients and positive symptoms partially mediate this association. The models built by peripheral inflammatory indices have a good median performance in predicting violent behavior in SCZ patients.

## Background

Schizophrenia (SCZ) is a debilitating mental illness, affecting approximately 1% of the world population. Its main clinical manifestations include positive symptoms (such as delusions, hallucination), negative symptoms (such as social withdrawal, apathy) and cognitive deficits [1]. It has been reported that SCZ patients have an increased risk of violence [2–4]. A recent meta-analysis including 51,309 patients with schizophrenia spectrum disorders from 15 countries has revealed that SCZ individuals were four times more likely to be engaged in violent behavior, compared to the general population [5]. The consequences of violent behaviors in SCZ not only place a burden on society but also contribute to stigma. Hence, it is of great importance to identify SCZ patients at a high risk of violence, so as to early adopt effective prevention strategies.

Machine learning (ML) belongs to a computational technique, and can compute an individual’s probability of exhibiting violence [6]. There have been several studies which combined ML and the risk factors associated with violence in SCZ to predict the risk of violence in SCZ patients [7–9]. However, the characteristics of the samples varied between studies, limiting the generalizability of the models to different populations [10]. In addition, neuroimaging markers were incorporated in the models for predicting violence in SCZ [11.12], but these markers are relatively expensive and not easy to obtain. Hence, the prediction of violent behavior in SCZ using the blood-based biomarkers may prove promising.

Accumulating evidence suggests that the changes of immune system play a crucial role in the pathogenesis of violent behavior [11, 12]. For instance, a study found soluble interleukin-1 Receptor II levels in cerebrospinal fluid were positively associated with aggression in human subjects [13]. Another study reported the plasma markers of oxidative stress were positively related to aggression in human subjects [11]. The levels of inflammatory indices were found higher in patients who were involved in crime than those not involved in crime [14]. Peripheral inflammatory indices such as the systemic immune inflammation index (SII) - the ratio between neutrophils (NEU) × platelet (PLT) and lymphocyte (LYM), the neutrophil lymphocyte ratio (NLR) - the ratio between NEU and LYM, the platelet-lymphocyte ratio (PLR) - the ratio between PLT and LYM and the monocyte lymphocyte ratio (MLR) - the ratio between monocyte (MON) and LYM are gaining prominence as the potential measures of inflammation due to being objective, easily accessible and cost-effective. These indices can reflect the balance between pro-inflammatory and anti-inflammatory cells [15, 16]. Some research has reported the changes of peripheral inflammatory indices in SCZ and positive associations between these indices and positive symptoms. For example, a retrospective study including 549 SCZ patients and 930 healthy controls found NLR and MLR values were increased in patients, compared to healthy subjects [17]. A 3-year retrospective study found elevations in MLR, NLR and SII in SCZ patients, compared to healthy controls [18]. A cross-sectional study found MLR, NLR, PLR and SII were positively correlated with PANSS positive scores [19]. In the literature, there is only one study that explored the relationship between peripheral inflammatory markers, aggression, impulsivity, and criminal behavior in SCZ patients [20]. However, they did not examine the associations between peripheral inflammatory markers, positive symptoms and violent behavior in SCZ individuals.

Hence, this study was aimed to examine the relationship between SII, NLR, PLR, MLR, positive symptoms in SCZ patients with and without violent behavior. Furthermore, on the basis of ML method, peripheral inflammatory markers were used to develop the predictive models for violence in SCZ patients.

## Materials

### Study participants

We performed a cross-sectional study in the general psychiatry ward of Hefei fourth people’s hospital in Anhui province, in the central and eastern part of China. A total of 160 SCZ inpatients who met the International Classification of Disease-10 (ICD-10) diagnostic criteria were recruited in the present study from April to December 2022 and divided into violent group with 66 patients and non-violent group with 94 patients, based on whether their Modified Overt Aggression Scale (MOAS) weighted total score was greater than 5 at admission. The inclusion criteria were as follows: minimum age of 18, capable of understanding the interview. Patients who met the following criteria were excluded from this study: mental retardation; autoimmune diseases, such as systemic lupus erythematosus, rheumatoid arthritis, type 1 diabetes, inflammatory bowel disease; acute or chronic inflammatory illnesses caused by surgery and severe infection; obesity; receiving treatment with anti-inflammatory or immunosuppressive medications within 6 months. The Ethics Committee of Anhui Medical University and Hefei Fourth People’s Hospital approved the study.

### Sample size calculation

The sample size was calculated using the formula: n=[Z^2^ * *p* *(1-*p*)]/e^2^. Z-score was set to 1.95 corresponding to a level of confidence (95%), *p* utilized the reported prevalence of violent behavior of SCZ patients in our previous research [9], and the allowable error value e was 0.08. Considering a rejection rate of 10%, the final sample size n was at least 154.

### Data collection

The trained psychiatrists used the form inserted in hospital electronic system to collect demographic and clinical data of all participants, including age, sex, smoking, alcohol use, education level, hospitalization frequency and duration of disease. Moreover, these demographic and clinical variables were considered to be the potential confounding variables, instead of being risk factors for violence in SCZ patients.

### Clinical assessment

Within 24 h after admission, Violent behavior and positive symptoms of all participants were evaluated using MOAS and Positive and Negative Syndrome Scale (PANSS), respectively. The MOAS composed of 4 items (verbal aggression, aggression against objects, physical aggression against oneself, and physical aggression against others) was adopted to assess the risk of violence in patients with SCZ [21]. When a MOAS weighted total score was greater than 5 at admission, violent behavior was defined. The positive subscale in PANSS was used for the evaluation of positive symptoms. This subscale consists of 7 items [22].

### Blood sample collection and analysis

The venous blood samples of all subjects were collected by the nurses in the morning of second day after admission and stored in EDTA tubes. All blood samples were sent to the laboratory center of our hospital for testing. An automatic blood analyser (LH 750, USA) was used for routine blood tests including white blood cell count (WBC), NEU, LYM, MON, eosinophilsl (Eos), basophils (Bas), red blood cell (RBC), hemoglobin (HGB) and PLT. We calculated SII, NLR, PLR and MLR using the following formulas. SII = NEU × PLT / LYM, NLR = NEU / LYM, PLR = PLT / LYM, MLR = MON / LYM.

### Model building process

The predictive models were established in R 4.0.5 software. Seven ML algorithms including support vector machine (svm), k-nearest neighbor (knn), random forest (rf), generalized linear model net (glmnet), rpart, penalized discriminant analysis (pda) and neural network (nnet) were applied in the establishment of predictive models. In order to facilitate replication, default parameters were used in all models. All participants were randomly divided into the training and the test sets, according to 1:2. In the training set, we employed tenfold cross-validation to control for overfitting. Through the method, all individuals were randomly divided into ten equal folds, in which nine folds were used for adjusting the parameters of predictive models through learning and the remaining fold for the selection of the best model. Ten rounds of training and validation were conducted. The test set is used to assess the performance of the final model. In this study, we used AUC value to evaluate the diagnostic performance of each model. A combination of four peripheral inflammatory biomarkers was used for the establishment of predictive models based on seven ML algorithms. Subsequently, the corresponding model of each peripheral inflammatory biomarker was developed using the optimal algorithm.

### Statistical analysis

SPSS software (version 16.0) was used for statistical analysis. Continuous variables were given as mean ± standard deviation (SD) or median with interquartile range (IQR), based on whether they were normal distribution. Then, the comparison of variables between groups was performed using Mann-Whitney U-test or independent samples t-test. The categorical variables were expressed as n(%) and comparison between groups was performed using chi-square test. We used general linear model (GLM) to compare the differences of inflammatory indices between groups, after controlling for the potential confounding variables. Spearman’s correlation was employed to examine the association between peripheral inflammatory biomarkers and PANSS positive scores. The difference method was employed to conduct a mediation analysis. We set peripheral inflammatory biomarkers as independent variables, positive symptoms as mediator variables, and violent behavior as dependent variable. The demographic and clinical variables with significant differences between groups were controlled as covariates in the mediation analysis. *P*<0.05 was considered to be statistically significant.

## Results

Demographic and clinical characteristics of subjects are shown in Table 1. A total of 160 SCZ patients were included in the present study and 66 patients exhibited violent behavior, with its prevalence being 41.25%. Age was decreased in the violent group compared to the non-violent group, whereas there were no significant differences in sex, smoking, alcohol use, education level, hospitalization frequency and the course of disease between groups. After controlling for age, covariance analysis showed neutrophils, monocytes, SII, NLR and MLR were significantly elevated in SCZ patients with violence, compared to those without violence (Table 2).

**Table 1:** Comparison of demographic and clinical characteristics between groups

Variables	violent group (66)	non-violent group (94)	t/χ2 value	*P* value
Age (year)	35.98 ± 12.43	40.70 ± 15.19	−2.15	0.033
Sex (%)				
Male	36 (54.55)	47 (50.00)	0.321	0.571
Female	30 (45.45)	47 (50.00)		
Smoking (%)				
yes	9 (13.64)	8 (8.51)	1.073	0.300
no	57 (86.36)	86 (91.49)		
Alcohol use (%)				
yes	9 (13.64)	9 (9.57)	0.641	0.423
no	57 (86.36)	85 (90.43)		
Education level (%)				
Primary school	13 (19.70)	18 (19.15)	3.36	0.186
Junior or senior high school	45 (68.18)	54 (57.45)		
College	8 (12.12)	22 (23.40)		
Hospitalization frequency	3.5(1, 5.25)	2(1, 6)	−0.17	0.863
Duration of disease (year)	12.91 ± 8.77	13.00 ± 10.71	−0.05	0.958
Positive symptom	29.64 ± 16.78	23.93 ± 15.17	2.22	0.028

**Table 2:** Analysis of laboratory parameters of the participants

Variables	violent group (66)	non-violent group (94)	F value	*P* value
WBC (10^9^ /L )	6.87 ± 1.86	6.31 ± 2.03	1.798	0.074
Neutrophils (10^9^ /L)	4.30 ± 1.68	3.68 ± 1.62	2.346	0.020
Lymphocytes (10^9^ /L)	1.94 ± 0.64	2.09 ± 0.82	−1.273	0.205
Monocytes (10^9^ /L)	0.50 ± 0.18	0.43 ± 0.15	2.737	0.007
Platelets (10^9^ /L)	207.23 ± 60.75	194.31 ± 53.30	1.424	0.156
SII	501.89 ± 337.88	359.23 ± 165.29	12.104	0.001
NLR	2.51 ± 1.62	1.89 ± 0.84	11.687	0.001
PLR	114.76 ± 45.38	102.26 ± 39.20	2.965	0.087
MLR	0.28 ± 0.12	0.22 ± 0.07	12.934	<0.001

Abbreviations: WBC, blood cell count; SII, systemic immune inflammation index, NLR, neutrophil lymphocyte ratio, PLR, platelet-lymphocyte ratio, MLR, monocyte lymphocyte ratio

After controlling for age, partial correlation analysis showed SII, NLR and MLR were positively associated with positive symptom in all subjects (Table 3).

**Table 3:** Associations of positive symptom with peripheral inflammatory biomarkers

Inflammatory indices	*r* value	*p* value
SII	0.202	0.012
NLR	0.259	0.001
MLR	0.291	<0.001
PLR	0.090	0.266

These associations were performed in all participants, controlling for age

Abbreviations: SII, systemic immune inflammation index; NLR, neutrophil lymphocyte ratio; PLR, platelet-lymphocyte ratio; MLR, monocyte lymphocyte ratio

After controlling for age, positive symptoms partially mediated the associations between NLR and MLR with violent behavior in SCZ patients (Table 4).

**Table 4:** The association of peripheral inflammatory biomarkers with violent behavior mediated by positive symptoms

Effects	SII	NLR	MLR
Controlled direct	0.003	0.376	6.197
Indirect	0	0.048	0.534
Total	0.003	0.424	6.731
Proportion eliminated	0	11.32%	7.93%

Abbreviations: SII, systemic immune inflammation index; NLR, neutrophil lymphocyte ratio; MLR, monocyte lymphocyte ratio

**Fig. 1:**
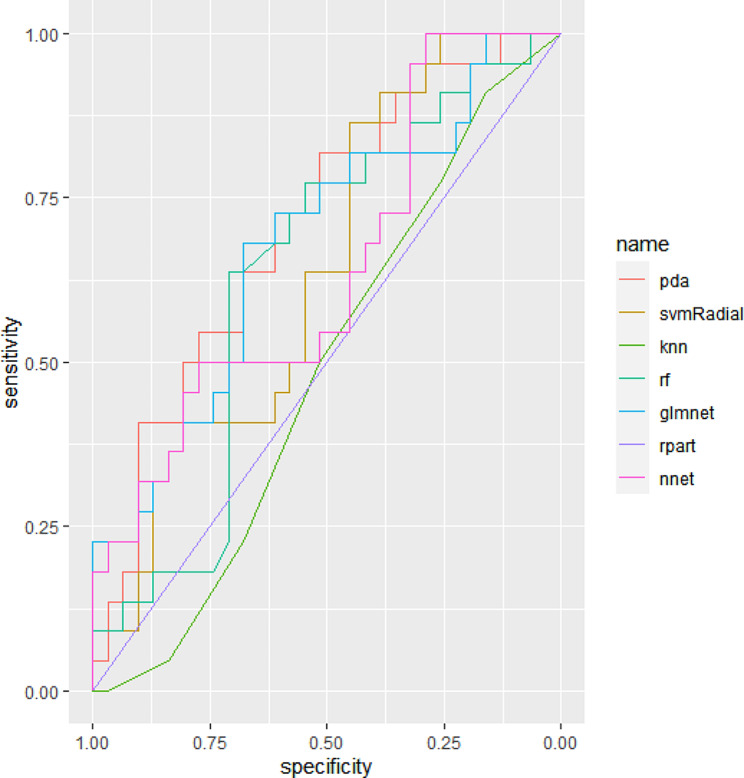
ROC curves for seven machine learning algorithms in identifying SCZ patients with violence. svm, support vector machine; knn, k-nearest neighbor; rf, random forest; glmnet, generalized linear model net; pda, penalized discriminant analysis; nnet, neural network

Compared to other algorithms, pda performed better and its AUC value was 0.7082 (Table 5; Fig. 1).

**Table 5:** Comparison of performance of seven algorithms

Algorithms	AUC	accuracy	kappa	sensitivity	specificity
svm	0.6408(0.4905–0.7910)	0.5849	0	0	1.0000
knn	0.4831(0.3287–0.6375)	0.4906	−0.0999	0.2273	0.6774
rf	0.6342(0.4793–0.7890)	0.5472	0.0290	0.3182	0.7097
glmnet	0.6789(0.5296–0.8282)	0.6604	0.2180	0.2272	0.9677
rpart	0.5000(0.5000–0.5000)	0.5849	0	0	1.0000
pda	0.7082(0.5665–0.8499)	0.6792	0.2977	0.4091	0.8710
nnet	0.6422(0.4881–0.7964)	0.6415	0.1806	0.2272	0.9355

Abbreviations: svm, support vector machine; knn, k-nearest neighbor; rf, random forest; glmnet, generalized linear model net; pda, penalized discriminant analysis; nnet, neural network; AUC, area under curve of ROC.

**Fig. 2:**
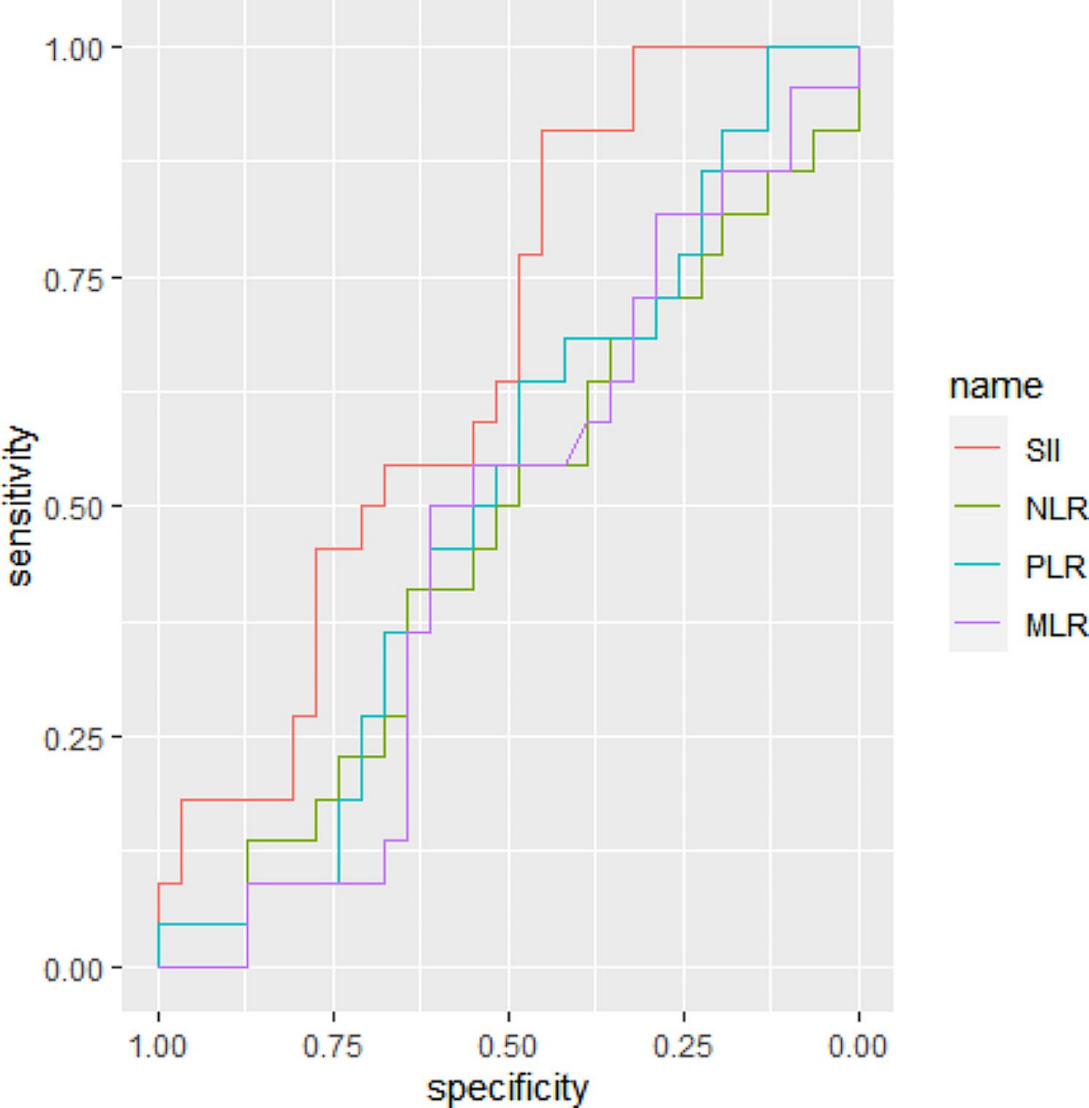
ROC curves for four peripheral inflammatory biomarkers in identifying SCZ patients with violence, based on pda. pda, penalized discriminant analysis

Based on pda, four inflammatory indices were used to develop the corresponding predictive models. SII had better performance than other indices, with its AUC value being 0.6613 (Table 6; Fig. 2).

**Table 6:** Comparison of performance of four inflammatory indices

Inflammatory indices	AUC	accuracy	kappa	sensitivity	specificity
SII	0.6613(0.5145–0.8081)	0.5293	0.0671	0.0909	0.9677
NLR	0.5849(0.4413–0.7186)	0.5198	0.0443	0.1364	0.9032
PLR	0.5117(0.3532–0.6703)	0.5472	−0.0426	0.0909	0.8710
MLR	0.5849(0.4413–0.7186)	0.5849	0.0443	0.1364	0.9032

Abbreviations: SII, systemic immune inflammation index; NLR, neutrophil lymphocyte ratio; PLR, platelet-lymphocyte ratio; MLR, monocyte lymphocyte ratio

## Discussion

The main findings of our study are as follows: the prevalence of violence in individuals with SCZ was 41.25%. SCZ patients with violence experienced more severe positive symptoms, and had the elevated SII, NLR and MLR values, in comparison to those without violence. After controlling for age, NLR and MLR were positively associated with positive symptoms in SCZ patients. Finally, based on seven ML algorithms, different inflammatory indices between groups (SII, NLR and MLR) were used for developing the predictive models for violence in SCZ. Among all algorithms, pda was found to have the best performance and its AUC was 0.7082.

Our results showed 41.25% of SCZ patients were found to have violent behavior. The finding is consistent with a study done by Huang et al. reporting 41.8% of community-dwelling patients with SCZ had violent behavior in china [23], but it is inconsistent with the rates of violent behavior reported in other studies. For instance, A cross-sectional comparative study showed 58% of SCZ patients had risk of violence [24]. The possible reason is the prevalence of violence in SCZ patients varies according to risk factors [25]. Despite inconsistent rates of violence, it is generally demonstrated that SCZ patients are more vulnerable to commit violence, compared to the general population.

In this study, there was a significance difference between violent and non-violent group regarding positive symptoms where patients with violence have more severe positive symptoms. The findings are in agreement with the results of several studies showing the contribution of positive symptoms to violence in SCZ [5, 23, 26, 27]. The possible explanation is positive symptoms especially persecutory ideations referred to as ‘threat-control override’ give patients a clear motivation for violence. As individuals diagnosed with mental illness always have compromised internal controls, When patients feel harmful and manipulative action that they believe to be directed against themselves, violence is committed as a defense of retaliation [28].

Another finding is that the increased SII, NLR and MLR values in individuals with violence, in relation to individuals without violence. It is well-established that the higher SII, NLR and MLR could reflect a dysregulation in the immune system, potentially leading to inflammation which may be implicated in violence in SCZ [29]. Nevertheless, this finding is inconsistent with the results of the only study which examined the associations between peripheral inflammatory markers and criminal behavior in SCZ individuals where they reported that there were no significant differences in NLR, MLR, SII and PLR values between patients with and without a crime history [11]. The conflicting findings could be explained by differences in sample size, the definition of violence and sex composition. In this study, a MOAS weighted total score greater than 5 was defined as violent behavior. However, the study by Suheda Kaya. et al. included SCZ patients involved in a crime. Additionally, the present study included male and female patients, but all participants were male in their research. In all participants, SII, MLR and NLR were found to be related to positive symptoms. Subsequently, the difference method was used to analyse the relationship of violent behavior, peripheral inflammatory markers and positive symptoms in SCZ patients, showing except SII, the associations between NLR and MLR with violence in SCZ were partially mediated by positive symptoms. The potential mechanism is that inflammation may result in neuronal toxicity, influence neurotransmitter synthesis, and cause damage to the cerebral microvascular blood flow, eventually resulting in the exacerbation of symptoms and the occurrence of violent behavior [30].

Studies using ML method and biological measures to predict violence in SCZ are limited [31, 32]. Our previous study based on structural magnetic resonance imaging (MRI) features using seven ML algorithms found that SVM as the optimal algorithm was capable of predicting violence in SCZ patients with an AUC of 0.841 [32]. Another similar study based on multimodal neuroimaging data found the final model combining gray matter volume, regional homogeneity and fractional anisotropy identified violent patients with SCZ with an AUC of 0.91 [31]. Compared their models, our best model with an AUC of 0.7082 was weaker. Considering that MRI data are expensive and quite difficult to obtain in some cases, peripheral inflammatory biomarkers are appropriate to clinical practice due to being relatively inexpensive and routinely measured. Additionally, four inflammatory indices were adopted to construct the predictive models using pda. The model developed by SII achieved better predictive powder than other indices, and its AUC was 0.6613, supporting the opinion that SII is a comprehensive indicator which reflects the balance between platelets, neutrophils, and lymphocytes and possesses more sensitive to showing inflammation than other indices [33].

There are a few limitations in our study. Firstly, this study belongs to the cross-sectional nature and fails to infer the causality of inflammation and positive symptoms. Secondly, the sample size of this study is limited, future studies with a large sample size is needed. Finally, the inflammatory status of patients may be influenced by other types of medicaiton treating concomitant diseases, such as, diabetes mellitus and hypertension.

This study is the first to investigate the role of peripheral inflammatory biomarkers in violence of SCZ. Our results indicate that inflammatory indices including SII, NLR and MLR are associated with positive symptoms and violence in SCZ patients. Inflammation may lead to violence by positive symptoms. Moreover, inflammatory indices effectively predict violence in SCZ patients and will facilitate individualized treatment and interventions to prevent violence in SCZ patients.

## Data Availability

The data that support the findings of this study are available from Hefei Fourth People’ Hospital but restrictions apply to the availability of there data, which were used under license for the current study, and so are not publicly available. Data are however available from the corresponding author upon reasonable request and with permission of Hefei Fourth People’ Hospital.

## Abbreviations

SCZ: Schizophrenia
MOAS: Modified overt aggression scales
PANSS: Positive and Negative Syndrome Scale
AUC: Area under curve of ROC
ICD-10: The International Classification of Disease-10
SVM: Support vector machine
ML: Machine learning
MRI: Magnetic resonance imaging
SII: Systemic immune inflammation index
NLR: Neutrophil lymphocyte ratio
PLR: Platelet-lymphocyte ratio
MLR: Monocyte lymphocyte ratio
WBC: Blood cell count
NEU: Neutrophils
LYM: Lymphocyte
MON: Monocyte
Eos: Eosinophilsl
Bas: Basophils
RBC: Red blood cell
HGB: Hemoglobin
PLT: Platelet
knn: k-nearest neighbor
rf: Random forest
glmnet: Generalized linear model net
pda: Penalized discriminant analysis
nnet: Neural network
